# Draft Genome Sequence of the Industrially Significant Bacterium Pseudomonas fluorescens ATCC 13525

**DOI:** 10.1128/MRA.01368-18

**Published:** 2018-11-01

**Authors:** M. J. Meier, R. M. Subasinghe, L. A. Beaudette

**Affiliations:** a Biological Assessment and Standardization Section, Environment and Climate Change Canada, Ottawa, Ontario, Canada; Georgia Institute of Technology

## Abstract

Pseudomonas fluorescens is a Gram-negative bacterium with versatile metabolic functions and potential industrial uses. We sequenced P. fluorescens strain ATCC 13525 with the goal of determining virulence factors and antibiotic resistance genes to predict the potential impacts on human and environmental health in the event of exposure.

## ANNOUNCEMENT

Pseudomonas fluorescens strain ATCC 13525 is on Canada’s domestic substances list (DSL) (a list of substances that were manufactured in, imported into, or used in Canada on a commercial scale prior to 1986, available at https://www.canada.ca/en/environment-climate-change/services/canadian-environmental-protection-act-registry/substances-list/domestic.html). P. fluorescens is a useful organism because of its versatile metabolism, ability to tolerate and biodegrade a wide range of substances, such as aromatic hydrocarbons, and metal tolerance (1–6). Specifically, it has applications in wastewater drains and treatment facilities, sewers, grease traps, and septic systems; it also has applications in the agriculture industry for pest control (7).

A glycerol stock of P. fluorescens strain ATCC 13525 (obtained from the ATCC) was used to inoculate a Difco nutrient agar plate for the isolation of single colonies. A single colony was then used to inoculate an overnight culture in Difco nutrient broth (37°C with shaking at 220 rpm), and total genomic DNA was isolated using the Wizard genomic DNA purification kit (Promega). Genomic DNA (1 µg) was enzymatically fragmented (10 min using the Ion Shear Plus reagents kit; Thermo Fisher Scientific) and size selected using the E-Gel SizeSelect agarose gel to select 300-bp fragments. We constructed a genomic library for sequencing using the Ion Xpress Plus DNA fragment library kit (Thermo Fisher Scientific) and sequenced on the Ion Torrent Personal Genome Machine (PGM) platform (Life Technologies).

We obtained 2,141,339 raw reads with an average length of 241 bp (516,853,298 total bases). Reads were processed using the EDGE bioinformatics platform, version 2.3.0 (8). Default parameters were used for quality trimming (with the addition of adapter sequence removal of the P1 and A adapter sequences), after which 1,986,147 reads with an average length of 257 bp remained (510,147,260 total bases). Assembly was performed using SPAdes version 3.11.1 (9), which produced 279 contigs, with an *N*_50_ of 57,275 bp (contig size range, 222 to 192,127 bp), and a total assembly size of 6,447,709 bp. The mean GC content was 60%. Mapping quality-controlled reads to the contigs using BWA-MEM (10) in EDGE revealed an average genome coverage of 78×. Contigs were annotated using the NCBI Prokaryotic Genome Annotation Pipeline (11), resulting in a total of 6,232 genes (5,205 coding genes).

We identified two antimicrobial resistance genes in this strain using the Antibiotic Resistance Genes Database (ARDB) through EDGE, an undecaprenyl pyrophosphate phosphatase (baca class, from the family NP_745006 [http://ardb.cbcb.umd.edu/cgi/search.cgi?db=L&field=ni&term=NP_745006]) and a resistance-nodulation-cell division transporter system (multidrug resistance efflux pump, mexab class, from the family YP_298289 [http://ardb.cbcb.umd.edu/cgi/search.cgi?db=L&field=ni&term=YP_298289]). A total of 75 virulence factors were identified, as follows: 37 adherence genes (32 flagella and 5 type IV pili biosynthesis genes), 18 antiphagocytosis genes (11 alginate biosynthesis and 7 alginate regulation), 12 secretion system genes (Hcp secretion island 1-encoded type VI secretion system), 4 iron uptake genes (pyoverdine), 2 regulation genes (GacS/GacA two-component system), 1 fibronectin-binding protein, and 1 biosurfactant gene (rhamnolipid biosynthesis). Industrial users of this microorganism can use this genome sequence to help establish safe work practices, and regulatory agencies can identify genes of interest that may apply to future risk assessments.

### Data availability.

This whole-genome shotgun project has been deposited in GenBank under the BioProject number PRJNA486371 (BioSample number SAMN09844436, accession number QVNA01000000) and the Sequence Read Archive under the accession number SRR7961511. This announcement describes the first version.

## References

[1] Workentine ML, Harrison JJ, Stenroos PU, Ceri H, Turner RJ 2008 *Pseudomonas fluorescens*’ view of the periodic table. Environ Microbiol 10:238–250. doi:10.1111/j.1462-2920.2007.01448.x. 17894814

[2] Rhodes ME 1959 The characterization of *Pseudomonas fluorescens*. Microbiology 21:221–263. doi:10.1099/00221287-21-1-221.

[3] Appanna VD, Gazsó LG, Pierre MS 1996 Multiple-metal tolerance in *Pseudomonas fluorescens* and its biotechnological significance. J Biotechnol 52:75–80. doi:10.1016/S0168-1656(96)01623-9.

[4] Barathi S, Vasudevan N 2001 Utilization of petroleum hydrocarbons by *Pseudomonas fluorescens* isolated from a petroleum-contaminated soil. Environ Int 26:413–416. doi:10.1016/S0160-4120(01)00021-6. 11392760

[5] Bugg T, Foght JM, Pickard MA, Gray MR 2000 Uptake and active efflux of polycyclic aromatic hydrocarbons by *Pseudomonas fluorescens* LP6a. Appl Environ Microbiol 66:5387–5392. doi:10.1128/AEM.66.12.5387-5392.2000. 11097918 PMC92472

[6] Caldini G, Cenci G, Manenti R, Morozzi G 1995 The ability of an environmental isolate of *Pseudomonas fluorescens* to utilize chrysene and other four-ring polynuclear aromatic hydrocarbons. Appl Microbiol Biotechnol 44:225–229. doi:10.1007/BF00164506.

[7] Health Canada, Environment Canada. 2015 Final screening assessment for *Pseudomonas fluorescens* ATCC 13525. Environment Canada, Ottawa, Ontario, Canada http://publications.gc.ca/pub?id=9.700327&sl=0.

[8] Li P-E, Lo C-C, Anderson JJ, Davenport KW, Bishop-Lilly KA, Xu Y, Ahmed S, Feng S, Mokashi VP, Chain PSG 2017 Enabling the democratization of the genomics revolution with a fully integrated Web-based bioinformatics platform. Nucleic Acids Res 45:67–80. doi:10.1093/nar/gkw1027. 27899609 PMC5224473

[9] Nurk S, Bankevich A, Antipov D, Gurevich AA, Korobeynikov A, Lapidus A, Prjibelski AD, Pyshkin A, Sirotkin A, Sirotkin Y, Stepanauskas R, Clingenpeel SR, Woyke T, McLean JS, Lasken R, Tesler G, Alekseyev MA, Pevzner PA 2013 Assembling single-cell genomes and mini-metagenomes from chimeric MDA products. J Comput Biol 20:714–737. doi:10.1089/cmb.2013.0084. 24093227 PMC3791033

[10] Li H, Durbin R 2009 Fast and accurate short read alignment with Burrows-Wheeler transform. Bioinformatics 25:1754–1760. doi:10.1093/bioinformatics/btp324. 19451168 PMC2705234

[11] Tatusova T, DiCuccio M, Badretdin A, Chetvernin V, Nawrocki EP, Zaslavsky L, Lomsadze A, Pruitt KD, Borodovsky M, Ostell J 2016 NCBI Prokaryotic Genome Annotation Pipeline. Nucleic Acids Res 44:6614–6624. doi:10.1093/nar/gkw569. 27342282 PMC5001611

